# Theranostic Calcium Phosphate Nanoparticles With Potential for Multimodal Imaging and Drug Delivery

**DOI:** 10.3389/fbioe.2019.00126

**Published:** 2019-06-04

**Authors:** Madhumathi Kalidoss, Rubaiya Yunus Basha, Mukesh Doble, T. S. Sampath Kumar

**Affiliations:** ^1^Medical Materials Laboratory, Department of Metallurgical and Materials Engineering, Indian Institute of Technology Madras, Chennai, India; ^2^Department of Biotechnology, Indian Institute of Technology Madras, Chennai, India

**Keywords:** multimodal imaging, calcium phosphate nanoparticles, drug delivery, antibacterial, image contrast, ion substitution, bone substitute, theranostics

## Abstract

Calcium phosphate (CaP) bioceramics closely resemble the natural human bone, which is a main reason for their popularity as bone substitutes. However, this compositional similarity makes it difficult to distinguish CaPs, especially in particulate form, from native bone by imaging modalities such as X-ray radiography, computed tomography (CT), and magnetic resonance imaging (MRI) to monitor the healing progress. External contrast agents can improve the imaging contrast of CaPs but can affect their physicochemical properties and can produce artifacts. In this work, we have attempted to improve the contrast of CaP nanoparticles *via* ion substitutions for multimodal imaging. Calcium-deficient hydroxyapatite (CDHA) nanoparticles with silver (Ag), gadolinium (Gd), and iron (Fe) substitution were prepared by a microwave-accelerated wet chemical process to improve the contrast in CT, T1 (spin–lattice), and T2 (spin–spin) MRI relaxation modes, respectively. Ag, Gd, and Fe were substituted at 0.25, 0.5, and 0.25 at.%, respectively. The ion-substituted CDHA (ICDHA) was found to be phase pure by X-ray diffraction (XRD) and Fourier transform infrared spectroscopy (FT-IR). Transmission electron microscopy (TEM) images showed that the ICDHA nanoparticles were platelet shaped and of 52 ± 2 nm length and 6 ± 1 nm width. The ICDHA showed high contrast in X-ray and CT compared to CDHA. The vibrating sample magnetometry (VSM) studies showed the ICDHA to exhibit paramagnetic behavior compared to diamagnetic CDHA, which was further confirmed by improved contrast in T1 and T2 MRI mode. In addition, the *in vitro* tetracycline drug loading and release was studied to investigate the capability of these nanoparticles for antibiotic drug delivery. It was found that a burst release profile was observed for 24 h with 47–52% tetracycline drug release. The ICDHA nanoparticles also showed *in vitro* antibacterial activity against *Staphylococcus aureus* and *Escherichia coli* due to Ag, which was further enhanced by antibiotic loading. *In vitro* biocompatibility studies showed that the triple-ion-substituted ICDHA nanoparticles were cytocompatible. Thus, the ion-substituted CDHA nanoparticles can have potential theranostic applications due to their multimodal image contrast, antibacterial activity, and drug delivery potential. Future work will be conducted with actual bone samples *in vitro* or in animal models.

## Introduction

Calcium phosphate (CaP) bioceramics are one of the most commonly studied bone substitutes used for the repair of bony defects and alveolar ridge augmentation, as ear implants and as bone graft extender in spinal fusion surgery (Vallet-Regi and Gonzalez-Calbet, 2004; Habraken et al., 2016). Their popularity in bone therapeutic application is due to their compositional similarity to bone mineral as well as excellent biocompatibility and bioactivity (Sampath Kumar and Madhumathi, 2016b). CaP has also been used as delivery carriers for antibiotics, anti-inflammatory agents, anticancer drugs, growth factors, proteins, and DNA for bone tissue engineering due to its high drug loading ability (Uskokovic and Uskokovic, 2011). Hydroxyapatite (HA), calcium-deficient hydroxyapatite (CDHA), tricalcium phosphate (TCP), and biphasic calcium phosphate (BCP) are some of the popularly used CaPs.

A major drawback of CaPs, especially in nanoparticulate forms, is the difficulty in differentiating them from native bone tissue by imaging modalities such as X-ray radiography and computed tomography (CT) (Ramanathan and Ackerman, 1999; Ventura et al., 2014). It is essential to follow the stability of the implanted biomaterial, its infiltration by bone cells, the new bone formation and bone remodeling to optimize the performance of such bone substitutes. Imaging offers a non-destructive way of quantitative and longitudinal analysis of the above parameters. CaPs have nearly the same X-ray absorption as natural bone due to their compositional similarity to bone mineral. Other imaging modes such as magnetic resonance imaging (MRI) have not been utilized to image hard tissue like bone and synthetic CaPs, as it is exceedingly difficult to obtain MR signals due to the short spin–spin relaxation times of the nuclei (Wu et al., 1999). It has been observed that even at a high resolution, there is lack of contrast between surrounding bone and synthetic CaP bone substitute (Beaman et al., 2006). In order to enhance the imaging contrast of CaP bioceramics, addition of contrast agents has been proposed. They include barium sulfate, tantalum oxide, strontium carbonate, gold, etc., for X-ray/CT imaging (Wang et al., 2007; Hoekstra et al., 2014; Mastrogiacomo et al., 2017) and gadolinium, iron, perfluorocarbon-based contrast agents, etc., for MR imaging (Wichlas et al., 2012; Mastrogiacomo et al., 2017; Nakamura et al., 2018). There are certain drawbacks associated with addition of such external contrast agents. For example, it has been shown that addition of CT and MRI contrast agents affects the setting time and mechanical properties of CaP and other bone cements (Tanomaru-Filho et al., 2012; Sun et al., 2013). Similarly, addition of external magnetic contrast agents like super paramagnetic iron oxide nanoparticles (SPIONS) can cause artifacts like blooming effects (Ventura et al., 2014).

Efforts toward improving the imaging of CaPs are currently focused on refining the current diagnostic techniques for better imaging and improving the inherent contrast of CaP bioceramics. Recent advancements in MRI are an example of the former approach. The development of ultra-short echo time and zero echo time techniques has made high-resolution bone imaging possible (Ventura et al., 2013; Dou et al., 2018). HA and other CaPs are highly amenable to functional modifications *via* ionic substitutions. Hence, toward the latter approach, contrast-enhanced CaPs have been developed by ionic substitutions (Qi et al., 2018). Since MRI and CT are two of the most common methods of imaging that, when combined together, can image both soft and hard tissues, it is very prudent to develop multimodal CT/MR contrast-enhanced CaP bioceramics.

Multimodal imaging refers to combining several imaging techniques by developing multifunctional contrast agents. Due to their multiple binding sites and functional groups, CaPs, in nanoparticulate form especially, have been widely tried as multimodal contrast agents for simultaneous diagnosis and therapeutic applications (nanotheranosis) (Qi et al., 2018). CaP nanoparticles have been used as contrast agents by conjugating with or encapsulating organic fluorophores, encapsulating gold nanoclusters, doping with elements [e.g., europium (Eu^3+^), manganese (Mn^2+^), gadolinium (Gd^3+^), iron (Fe^3+^), neodymium (Nd^3+^), terbium (Tb^3+^), dysprosium (Dy^3+^), etc.], conjugating with quantum dots, radiolabeling with samarium (^153^Sm), indium (^111^In), lutherium (^177^Lu), technetium (^99m^Tc), etc., for imaging modalities including fluorescence, CT, MR, and nuclear imaging (Qi et al., 2018). HA nanoparticles exhibiting paramagnetism, near-infrared (NIR) fluorescence, and X-ray absorption by Eu^3+^ and Gd^3+^ doping have been developed by Ashokan et al. (2010). They had also developed CaP nanoparticles doped with both indocyanine green (ICG) and gadolinium (Gd^3+^), and labeled with ^99m^Tc-MDP for combined optical, magnetic, and nuclear imaging (Ashokan et al., 2013). The synthesis of Gd^3+^-doped HA labeled with ^153^Sm for serving as a dual-modal probe for SPECT and MRI is another example for multimodal imaging using CaPs (Liu et al., 2014). Modified CaPs with therapeutic agents can be used to achieve disease diagnosis by enhancing the imaging contrasts combined with chemotherapy/gene therapy/hyperthermia therapy (HTT)/photodynamic therapy (PDT)/radiation therapy (RT), etc. (Qi et al., 2018). For example, Eu^3+^/Gd^3+^ dual-doped HA have been used for simultaneous photoluminescent and magnetic imaging along with drug delivery application (Chen et al., 2011). Folic-acid-functionalized, Gd^3+^-doped HA that functions as a theranostic system with MR imaging and production of HA-^159^Gd-^32^P radioisotope by neutron activation for active targeting of osteosarcomas has been developed (Cipreste et al., 2016). Similarly, HA doped with both Fe^2+^ and Fe^3+^ ions has been developed for theranostic applications (Tampieri et al., 2012). The Fe-doped HA exhibited intrinsic magnetization and can be used for multiple applications including hyperthermia treatment as well to develop new magnetic ceramics for enhanced bone regeneration. Tseng et al. (2014) have reported that ^111^In-labeled lipid CaP nanoparticles preferentially accumulated in the lymph nodes *via* lymphatic drainage, which were used for SPECT/CT imaging-guided lymphatic gene delivery.

In the current work, a multimodal CaP drug delivery system with intrinsic antibacterial activity and enhanced contrast for CT/MR imaging modalities has been attempted by ion substitution. The CDHA of Ca/P ratio 1.61 was chosen for triple ion substitutions based on our previous results where it was found to be superior to HA as a drug-releasing resorbable bone substitute for the prevention and treatment of bone infections (Madhumathi and Sampath Kumar, 2014). Our preliminary work has shown that silver and gadolinium substitution into the HA crystal structure has resulted in significant CT and T1 MR contrast (Madhumathi et al., 2014). Iron (Fe^3+^) with its large magnetic moments is used as a contrast agent in T2 relaxation mode of MRI (Chandra et al., 2012). Hence, the substitution of Fe along with Ag and Gd in CDHA for the additional T2 MR contrast will be studied. The contrast enhancement provided by the triple-ion-substituted CDHA nanoparticles will be characterized through X-ray, CT imaging, and MR imaging modes. In addition, the *in vitro* tetracycline loading and release profile, antibacterial activity, and biocompatibility of the substituted CDHA nanoparticles will also be evaluated.

## Materials and Methods

### Materials

Analytical grade calcium nitrate Ca(NO_3_)_2_·4H_2_O, diammonium hydrogen phosphate (NH_4_)_2_HPO_4_, silver nitrate Ag(NO_3_), gadolinium oxide Gd_2_O_3_, ferric nitrate nonahydrate Fe(NO_3_)_3_·9H_2_O, and ammonia (30% GR) were purchased from MERCK, India. The drug tetracycline was purchased from Sigma Aldrich, India.

### Synthesis of Pure CDHA Nanocarriers

Pure CDHA of Ca/P ratio 1.61 was prepared as reported earlier through a microwave-accelerated wet chemical reaction using Ca(NO_3_)_2_·4H_2_O and (NH_4_)_2_HPO_4_ as precursors (Siddharthan et al., 2004). The reaction was carried out at pH 10 using ammonia with stirring. The precipitate formed was subjected to microwave irradiation in a microwave oven (BPL India, 2.45 GHz, 800 W power) for 20 min, after which it was washed with distilled water and dried. The dried samples were then powdered using an agate mortar and pestle.

### Synthesis of Triple-Ion-Substituted ICDHA Nanocarriers

The triple-ion-substituted CDHA was prepared by adding Ag, Gd, and Fe precursors along with Ca(NO_3_)_2_4H_2_O and (NH_4_)_2_HPO_4_ using the same microwave-accelerated wet chemical method mentioned in the section Synthesis of Pure CDHA Nanocarriers. The amount of precursors was calculated by fixing Ag^+^ concentration at 0.25 at.%, Fe^3+^ at 0.25 at.%, and Gd^3+^ at 0.5 at.%, restricting the total substitution to 1 at.%. The (Ca+Ag+Gd+Fe)/P ratios were maintained at 1.61. The sample was labeled as ICDHA.

### Material Characterization

The phase and crystallinity of the nanocarriers were characterized by X-ray diffraction method (XRD, Bruker D8 DISCOVER, USA) using Cu Kα radiation (λ **=** 1.54 Å). The diffraction patterns were recorded at a scanning rate of 1 step/s with a step size of 0.1°/step. The functional groups present in the CDHA and ICDHA nanocarriers were analyzed by Fourier transform infrared spectroscopy (FT-IR, Perkin-Elmer Spectrum Two, Germany) in the attenuated total internal reflectance (ATR) mode. The FT-IR spectra were collected from the range 4,000**–**510 cm^−1^. The elemental composition of ICDHA was studied by scanning electron microscopy (SEM, FEI Quanta 200, Netherlands) using energy dispersive X-ray (EDS) analysis. The particles were dispersed in distilled water and ultrasonicated for 5 min. The dispersed nanoparticles were then sprayed on a cylindrical metal stub using an adhesive carbon tape. The surface was then dried and coated using gold for 2 min to improve the conductivity of the sample. Transmission electron microscopy (TEM, Philips CM20, Netherlands) was used to identify the size and morphology of the prepared nanocarriers. For TEM analysis, the apatite particles were dispersed in ethanol and ultrasonicated for 15 min. The dispersed samples were coated on carbon-coated copper grids, dried, and examined under TEM operated at 120 kV. The surface area measurements of the samples were obtained at the temperature of liquid nitrogen using the Brunauer**–**Emmett**–**Teller (BET) N_2_ adsorption/desorption isotherm method (Sorptomatic 1990, USA). The magnetic properties of the nanocarriers were evaluated using vibrating sample magnetometry (VSM) at room temperature in an applied magnetic field of **±** 1.5 T (Lakeshore VSM 7410, USA).

### *In vitro* Imaging Studies

The image contrast properties of the ion-substituted samples were studied using radiography (X-ray) and CT imaging. The powder samples were weighed and compressed into pellets of equal weight (200 mg) and 1 cm diameter. The X-ray radiographs were taken in a high-frequency X-ray generator (Siemens Multiphos 15, USA) along with aluminum standards of 1**–**5 mm thickness to differentiate the contrast. For CT scan, the pellets were placed inside a phantom model and imaged using a six-slice spiral CT (Philips Ingenuity Core128, USA) in a coronal section. For MR imaging, the nanocarriers were dispersed in distilled water. The MRI studies of triple-ion-substituted CDHAs were carried out by dispersing them in distilled H_2_O at different concentrations (10**–**200 mM) and imaged in both T1 and T2 mode using a preclinical MR system (Bruker Biospec, France) of 4.7 T.

### Drug Loading and Release

The drug loading and release studies were carried out from the ion-substituted samples using tetracycline following the procedure described earlier (Madhumathi and Sampath Kumar, 2014). About 5 mg of the drug was dispersed in 50 ml of suitable medium like ethanol or phosphate buffer solution (PBS). The CDHA and ICDHA nanocarriers (50 mg) were added to the drug-dispersed solution and placed in a constant temperature water bath at 37°C for 24 h. After 24 h, 2 ml of supernatant was removed for estimation of drug concentration using UV**–**Vis spectroscopy (Lambda 35, Perkin-Elmer, USA). The samples were centrifuged, filtered, and dried at room temperature for 24 h.

The amount of drug loaded onto the carriers was determined by the following equation:

(1) Drug loading %=(Ic−Fc)÷Ic×100

Where Ic and Fc are the initial and final concentration of drug in PBS.

The release study was performed by dispersing 10 mg of drug-loaded carriers in 10 ml of PBS of pH 7.4. The samples were placed in a constant temperature water bath at 37°C. The concentration of drug released was estimated by removing 2 ml of supernatant and replaced by fresh PBS at various time intervals. The drug release profile was determined by measuring the absorbance values at different time intervals (Fc) from the initial loaded concentration (Ic). All the experiments were performed in triplicate.

### *In vitro* Antibacterial Studies

The drug-loaded CDHA and ICDHA samples (10 mg) were added to 9 ml of nutrient broth. Pure and ion-substituted samples without drug were taken as controls. The suspensions were then inoculated with 1 ml of *Staphylococcus aureus* and *Escherichia coli* bacterial cultures and incubated at 37°C for 24 h with shaking. The antibacterial efficacy of the samples was determined from the optical density (OD) of the cultures at 600 nm using the following equation:

(2) $$\begin{array}{l} \text{Bacterial reduction }\%=\{1-\left(\text{sample OD }÷\text{ control OD}\right)\\ \;\times 100\} \end{array}$$

### *In vitro* Biocompatibility Studies

The biocompatibility of the drug-loaded triple-ion-substituted nanoparticulate system was tested against Swiss 3T3 fibroblast cells (NCCS, Pune) by the colorimetric MTT [3-(4, 5-181dimethylthiazole-2-yl)-2,5-diphenyl tetrazolium bromide] assay for 48 h. The Swiss 3T3 fibroblast cells were grown to confluence with Dulbecco's modified Eagle's medium (DMEM) supplemented with 10% fetal bovine serum (FBS) and 1% 100 × antibiotic–antimycotic liquid and incubated at 37°C with 5% carbon dioxide in a CO_**2**_ incubator (Astec, Japan). The cells were then trypsinized and counted with a hemocytometer (Marienfeld, Germany). They were then diluted at 5,000 cells per well, seeded in 96-well plates, and cultured for 24 h. Ten milligrams of the samples was suspended in 1 ml of DMEM and incubated at 37°C for 24 h. The media in the 96-well plates was then replaced with 100 μl of the supernatant from the CDHA samples and again incubated for 24 h. Twenty microliters of 5 mg/ml MTT was added to each well and incubated for 4 h. The purple formazan precipitate formed selectively by live cells was solubilized in dimethyl sulfoxide (DMSO), and the absorbance was measured at 570 nm using a multimode plate reader (EnSpire, Perkin-Elmer, Singapore). The percentage of viable cells was calculated as the percentage relative to the control (standard polystyrene tissue culture plates) using the following equation:

(3) Cell viability %=(Sample OD÷Control OD)×100

### Statistical Analysis

The values are expressed as mean ± SD. Statistical analysis was performed using one- and two-way ANOVA wherever applicable with a *p*-value < 0.05 considered statistically significant.

## Results

### Material Characterization

**Figure 1:**
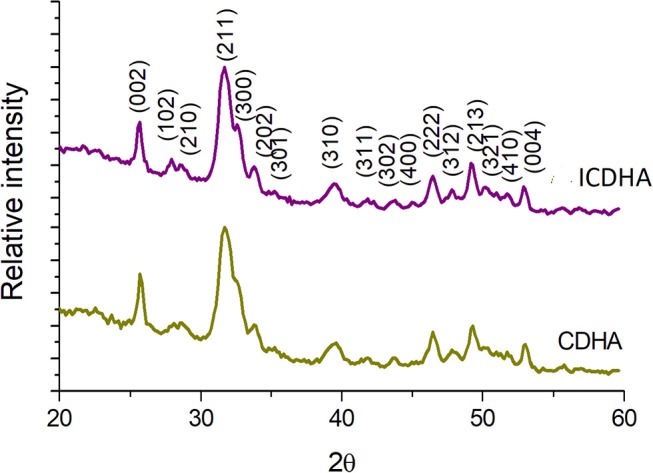
XRD pattern of pure CDHA and triple-ion-substituted CDHA (ICDHA).

The XRD pattern of pure CDHA and ICDHA is shown in Figure 1. The ion-substituted samples exhibit peaks similar to pure HA (JCPDS 09-432) and also revealed no secondary phases other than that of HA, suggesting ionic substitution in CDHA lattice. The cell parameters and cell volume calculated using “Unitcell” software and the crystallite size calculated using Scherrer's formula from XRD spectra are listed in Table 1. The ICDHA sample also showed larger cell parameters and a crystallite size of 49 nm when compared to pure CDHA with a crystallite size of 31 nm.

**Table 1:** List of cell parameters, cell volume, and crystallite size obtained from XRD and TEM results.

**Samples**	**Ion substitution (at.%)**	**Cell parameters (Å)**		**Cell volume (Å)^**3**^**	**Crystallite size (nm)**	
		***a***	***c***		**XRD**	TEM (L^*^W)
CDHA	–	9.371	6.862	505	31	(40 ± 3)^*^(5 ± 3)
ICDHA	0.25 Ag 0.5 Gd 0.25 Fe	9.470	6.860	533	49	(52 ± 2)^*^(6 ± 1)

**Figure 2:**
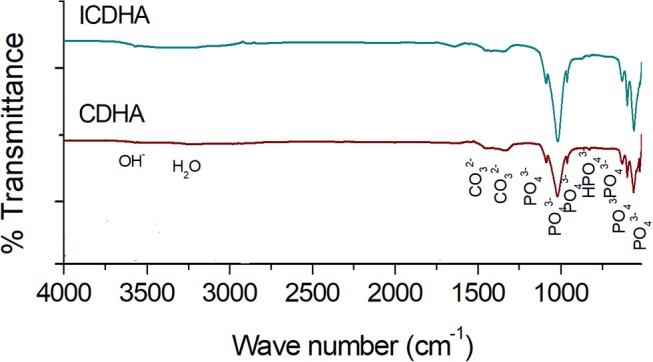
FT-IR spectra of pure CDHA and triple-ion-substituted CDHA (ICDHA).

The FT-IR spectra of pure and triple-ion-substituted CDHA are shown in Figure 2. All the vibration bands of ion-substituted CDHA correspond to that of the characteristic bands of pure CDHA. The peaks observed include lattice OH (3,573 cm^−c^); vibrational OH (634 cm^−v^); phosphate bands at 470, 565, 603, 962, 1,035, and 1,090 cm^−c^; carbonate (1,457 and 1,423 cm^−c^); and lattice water (3,422 cm^−c^). Additional ${\text{HPO}}_{4}^{2-}$ peak at 874 cm^−p^ was also observed from ion-substituted CDHA samples.

**Figure 3:**
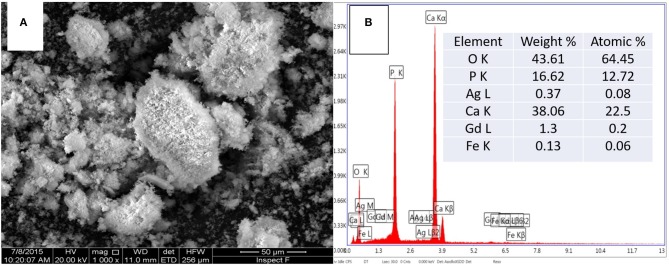
**(A)** SEM image and **(B)** corresponding EDS spectra of ICDHA.

**Figure 4:**
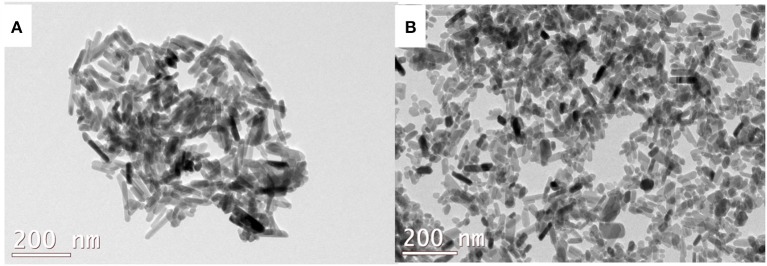
TEM image of **(A)** CDHA and **(B)** ICDHA.

**Figure 5:**
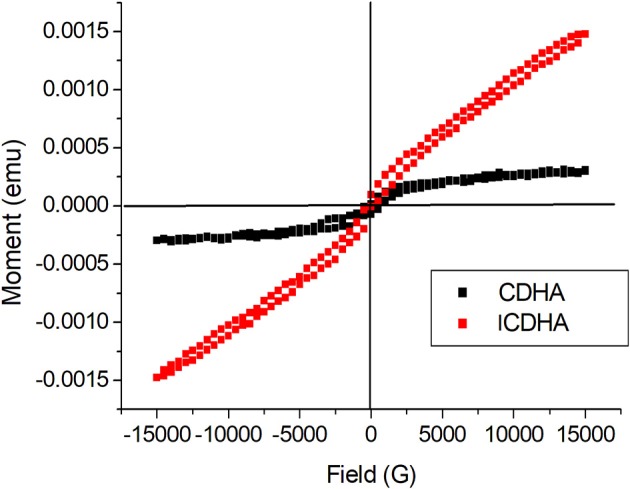
VSM graph showing magnetization properties of CDHA and ICDHA.

Figure 3 shows the SEM image and corresponding EDS spectra of ICDHA. It can be seen that ICDHAs appear as micron-sized aggregates in Figure 3A. The EDS spectra show the presence of Ag, Gd, and Fe in the samples in addition to Ca and P. The typical TEM images of triple-ion-substituted samples can be seen in Figure 4. The TEM image of ICDHA samples showed large nanoparticles with acicular morphology. The particle sizes calculated from TEM micrographs using ImageJ image analysis software are listed in Table 1. The surface areas of CDHA and ICDHA samples from BET studies were 58 and 45 m^2^/g, respectively. Figure 5 shows the results of VSM studies presenting the magnetization properties before and after ion substitutions in CDHA nanocarriers. It can very well be seen that the diamagnetic CDHA nanoparticles showed paramagnetic behavior on ion substitution.

### X-ray, CT, and MR Imaging Studies

**Figure 6:**
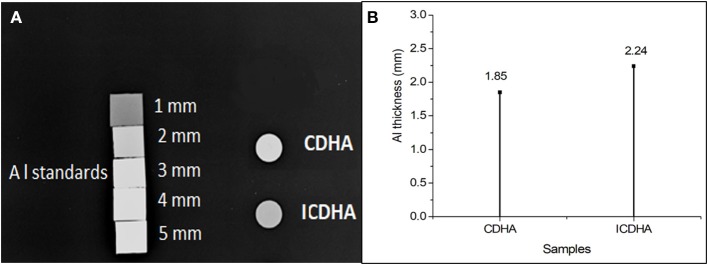
**(A)** X-ray and **(B)** grayscale value graph of CDHA and ICDHA.

**Figure 7:**
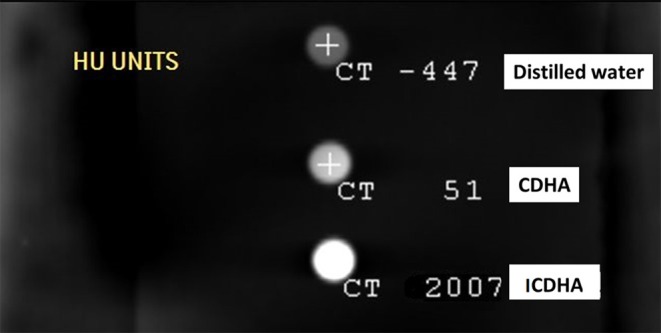
CT contrast of ICDHA samples compared with CDHA in HU (Hounsfield Units).

The X-ray radiographical image of the pellets of ICDHA in comparison to CDHA is shown in Figure 6A. It can be seen that the ICDHA pellet exhibited visibly higher contrast than pure CDHA. To further quantify the contrast (grayscale value), the pellets of the samples were compared along with aluminum (Al) standards of varying thickness (1–5 mm) as can be seen in Figure 6A. ImageJ software was used to quantify the grayscale value. Figure 6B shows the grayscale value graph corresponding to the X-ray image. The graph was obtained by plotting the contrast of the pellets (*x*-axis) with respect to the contrast of Al standards (*y*-axis). The graph shows that the contrast of ICDHA is comparable to that of 2.24 mm Al thickness while that of CDHA can be compared to 1.8-mm Al thickness. The substitution of elements with high density like Ag^+^ (10.49 g/cm^3^), Gd^3+^ (7.90 g/cm^3^), and Fe^3+^ (7.86 g/cm^3^) greatly increased the radiopacity of ICDHA compared to CDHA. The CT scan radiograph with ICDHA and CDHA samples is shown in Figure 7. The contrast values in CT are quantified in Hounsfield Units (HU). While both CDHA and ICDHA showed greater contrast than distilled water (control), ICDHA (2007 HU) exhibited much superior contrast than CDHA (51 HU).

**Figure 8:**
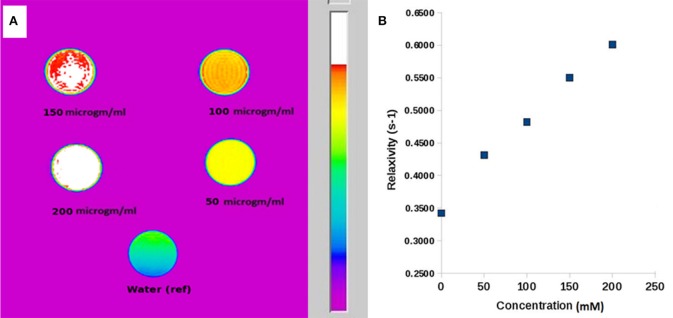
**(A)** T1-weighted MR image of ICDHA substituted samples dispersed at different concentrations in distilled water and **(B)** longitudinal relaxivity vs. concentration graph of ICDHA.

The MR contrast images of ICDHA in T1 and T2 mode are shown in Figure 8. As observed in Figure 8A, the T1 MR contrast increased for the ICDHA samples with increasing concentrations. The color bar adjacent to the MR image shows the color change from bottom to top according to increase in contrast. Significant T1 contrast was observed at a concentration of 50 μg/ml and increased up to the concentration of 200 μg/ml. Figure 8B shows the concentration vs. longitudinal relaxivity (r1) plot for ICDHA samples. The r1 was calculated according to the equation:

(4) $$\begin{array}{l} {\left(1÷T_{1}\right)}_{obs}={\left(1÷T_{1}\right)}_{dia}+r1M \end{array}$$

where (1/T_1_)_obs_ is the observed relaxation rate in the presence of ICDHA, (1/T_1_)_dia_ is the diamagnetic relaxation rate in the absence of nanoparticles, and M is the molar concentration (Ashokan et al., 2010). The T1 relaxivity increased with an increase in the ICDHA nanocarrier concentration. The maximum r1 observed for ICDHA nanocarriers was 0.61 mM^−1^ s^−1^ for 200 μg/ml.

**Figure 9:**
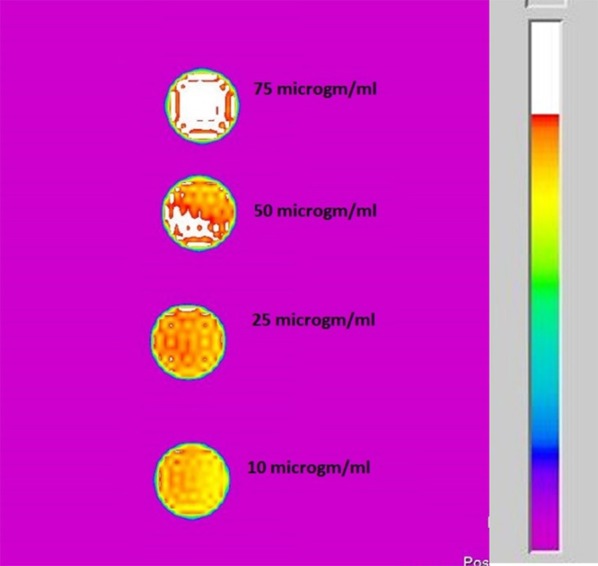
T2-weighted MR image of ICDHA substituted samples dispersed at different concentrations in distilled water.

Figure 9 shows the T2 MR contrast for different concentrations of the ICDHA samples (10–200 μg/ml). The T2 images were obtained by pulse sequence spin-echo technique with TR/TE = 4,000/60 ms and FOV as 8 × 8 cm^2^. Mild T2 contrast was observed at 10 μg/ml concentration and increased for up 75 μg/ml concentration with no further increase. The contrast obtained in T2 mode was much lower than that of the T1 mode, and so the T2 relaxivity graph was not plotted.

### Drug Loading and Release Studies

**Figure 10:**
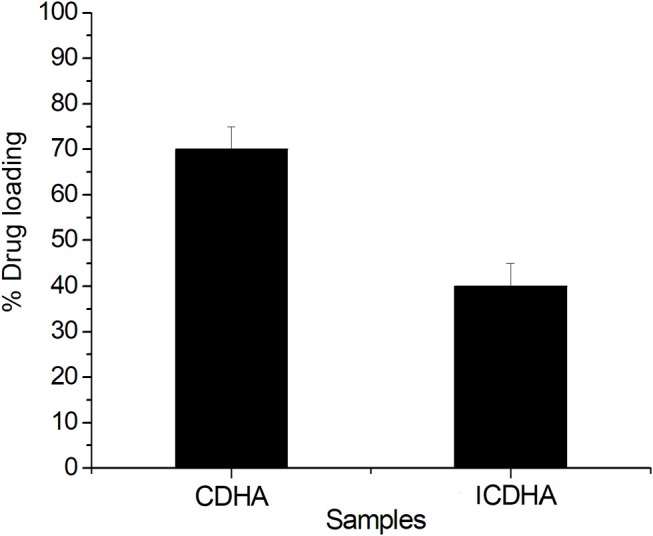
Loading profile of tetracycline from ion-substituted CDHA (ICDHA) compared to pure CDHA (*n* = 3; data shown as mean ± SD; *p* < 0.05, one-way ANOVA).

The drug loading and release studies were carried out using the tetracycline antibiotic. Figure 10 shows the drug loading percentage of ion-substituted apatites compared with pure samples. ICDHA showed a lower antibiotic loading (~40–55%) compared to CDHA (65–70%). This may be attributed to the lower surface area of ICDHA as well as due to the replacement of Ca^2+^ ions with other ions that might affect the drug binding.

**Figure 11:**
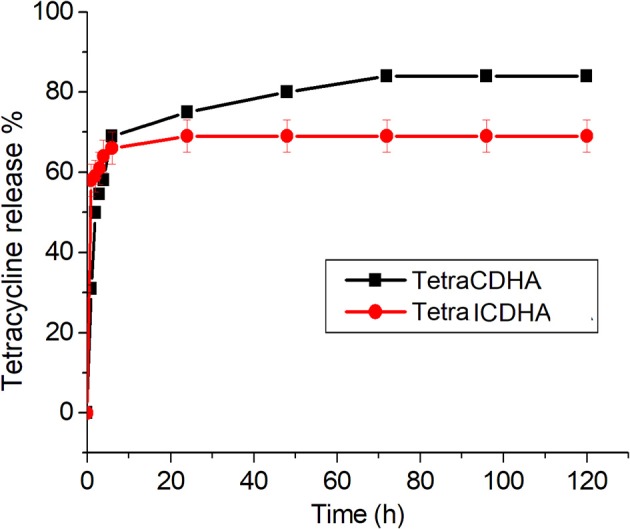
Tetracycline release profile from ICDHA compared to that of CDHA (*n* = 3; data shown as mean ± SD; *p* < 0.05, one-way ANOVA).

The drug release profile of tetracycline from CDHA and ICDHA, however, looks similar (Figure 11). The ion substitutions caused a minor change in the drug release profiles. The ICDHA samples showed a burst release of tetracycline with maximum release at 24 h, and the maximum release was lower than CDHA, which can be related to the changes in particle size as well as the non-stoichiometric nature of ion-substituted apatites.

### *In vitro* Antibacterial Studies

**Figure 12:**
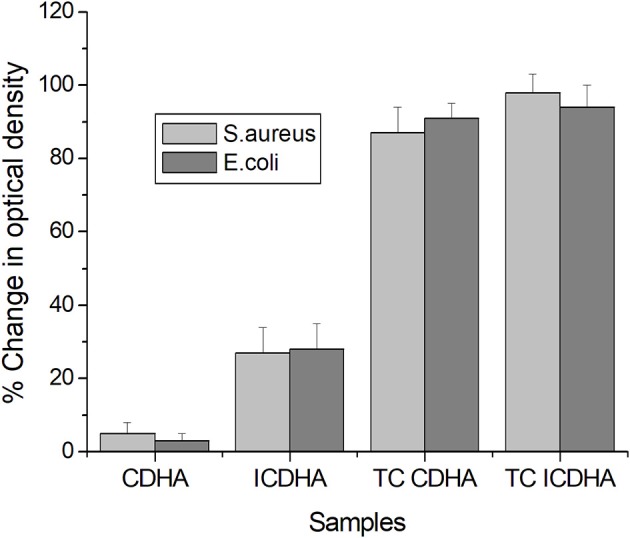
Antibacterial activity of pure and tetracycline-loaded CDHA and ICDHA (*n* = 3; data shown as mean ± SD; *p* < 0.05, one-way ANOVA).

The results of the antibacterial studies of ion-substituted apatites against *S. aureus* and *E. coli* are shown in Figure 12. Although pure CDHA was not antibacterial, the ICDHA showed antibacterial activity between 20 and 25%, which can be attributed to the presence of Ag^+^. The antibacterial activity further increased with tetracycline loading as expected. All tetracycline-loaded samples showed >90% activity against both bacterial strains. The ion-substituted tetracycline-loaded ICDHA showed the highest antimicrobial activity against *S. aureus*, which is known to cause osteomyelitis and prosthetic joint infection when introduced through trauma, surgery, direct colonization from a proximal infection, or systemic circulation (Sampath Kumar and Madhumathi, 2016a).

### *In vitro* Biocompatibility Studies

**Figure 13:**
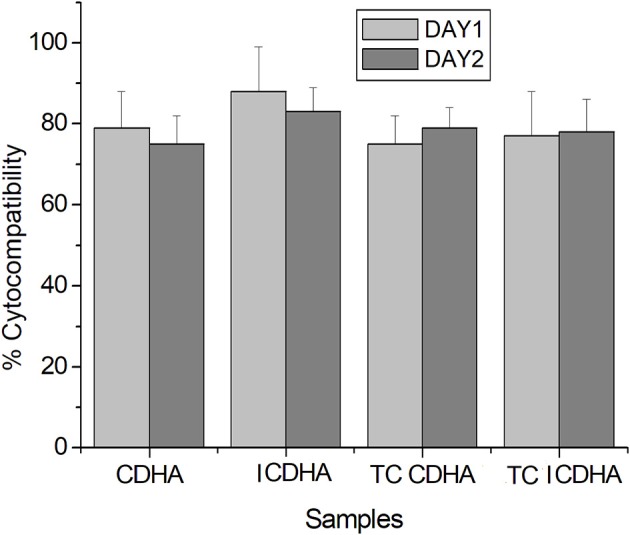
Biocompatibility of pure and tetracycline-loaded samples against Swiss 3T3 fibroblast cells by MTT assay for 24 and 48 h; TC, tetracycline (*n* = 3; data shown as mean ± SD; *p* < 0.05, two-way ANOVA).

The MTT assay results of pure and drug-loaded ICDHA apatites on Swiss 3T3 fibroblast cells are shown in Figure 13. It can be seen that all samples including the antibiotic-loaded CDHA and ICDHA samples showed 80–100% cell viability on Day 1 and 75–85% cell viability on Day 2. The high cell viability in ion-substituted samples may be due to the low total ionic substitution of ~1%.

## Discussion

Regeneration and healing of bone tissue post trauma, surgery, or infection often require the implantation of a natural bone graft or synthetic bone substitute. While the ability to aid and promote new bone formation is the main emphasis in developing such bone substitutes, it is also desirable to focus on other beneficial properties such as drug delivery ability, antibacterial activity, and optimal image contrast. In our previous studies, nanoparticulate bone substitutes with the abovementioned properties have been developed through approaches of drug loading and/or ion substitutions (Madhumathi and Sampath Kumar, 2014; Sampath Kumar et al., 2015). It was found that the substitution of ions such as Ag^+^, Gd^3+^, etc. imparted antibacterial activity and greater image contrast in CT and T1 MR modes.

Conventional MRI contrast agents enhance contrast either in T1 mode (bright signal) or T2 mode (dark signal). Recent research in the field of MR imaging focuses on the challenge to develop contrast agents that enhance both dual T1 and T2 MRI contrast. These T1/T2 contrast agents are designed for a single instrument and assumed to have advantages such as signal reliability and lack of image mismatch, which can occur when using different contrast agents or different instruments (Shin et al., 2015). An overlay of the T1 and T2 signals can help distinguish the targets from the surroundings. Dual T1/T2 imaging has been achieved through complex routes such as conjugation of T1 and T2 elements [e.g., covalently attaching Gd-DTPA with dopamine-coated iron oxide nanoparticles *via* isothiourea linkage, manganese (Mn)-embedded iron oxide nanoparticles, etc.] and magnetically decoupled T1–T2 dual mode contrast agents [e.g., Mn-doped iron oxide (MnFe_2_O_4_) nanoparticles as the core T2 contrast material and Gd_2_O(CO_3_)_2_ as the shell T1 contrast material separated by non-magnetic silica layer] (Bae et al., 2010; Choi et al., 2010; Huang et al., 2014). Since the apatitic structure allows for flexible ion substitution, we aimed to develop CDHA nanoparticles capable of simultaneous T1 and T2 MR contrast along with CT by substituting both Gd^3+^ and Fe^3+^ ions. One of the main criteria in deciding the percentage of Ag^+^, Gd^3+^, and Fe^3+^ ion substitutions in CDHA was biocompatibility, and the total ion substitution was restricted to 1 at.%. Previous reports have shown that the Ag^+^ and Gd^3+^ ion substitutions in HA have been found to be biocompatible at up to 0.5 and 4.4 at.%, respectively (Ramesh babu et al., 2007; Getman et al., 2010). Hence, the silver concentration was fixed at 0.25 at.%, the Gd^3+^ concentration was restricted to 0.50 at.% allowing Fe^3+^ substitution of 0.25 at.%, and the maximum combined substitution was restricted to 1 at.%.

The synthesized ICDHA samples were found to be phase pure as observed from XRD pattern and FT-IR spectra. The EDS spectra showed the presence of Ca, P, Ag, Gd, and Fe ions in the ICDHA samples. TEM image of ICDHA samples showed them to be of similar morphology but larger sized compared to CDHA. The ICDHA samples showed a greater X-ray and CT contrast than CDHA. ICDHA also showed a greater magnetic moment in VSM than pure CDHA. A dual T1/T2 imaging ability was observed with the ICDHA, although the contrast enhancement obtained in T1 mode was much higher than that in T2 mode. The relaxivity plot for the T2 mode was not obtained due to the low T2 contrast probably owing to the low at.% of Fe^3+^ substitution compared to Gd^3+^. However, a dual T1/T2 contrast using very low ionic substitution was demonstrated. Drug delivery studies using tetracycline were performed to observe any modification in the antibiotic binding and release kinetics due to ion substitution in ICDHA. The studies showed a distinct reduction in the loading and a tendency toward burst release for these samples compared to pure CDHA. While the low loading may be attributed to the lower surface area of ion-substituted apatites and loss of Ca^2+^ binding sites, the burst release in ICDHA can be attributed to the release of drugs adsorbed on the surface. The antibacterial studies on *S. aureus* and *E. coli* showed a significant antibacterial activity of ICDHA due to Ag^+^ even without antibiotic loading. With antibiotic binding, the antibacterial activity increased to >95%. Due to the low total ionic substitution of 1 at.%, both samples showed high biocompatibility for up to 48 h even with drug loading. Further *in vivo* studies can validate the above observations, suggesting that ICDHA samples can act as biodegradable theranostic nanocarriers of antibiotics with inherent CT/T1/T2 MR contrast and antibacterial activity.

## Conclusion

In the present study, ion-substituted CDHA nanoparticles with antibacterial activity and enhanced CT/T1/T2 MR image contrast for theranostic applications were developed. Ions such as Ag^+^, Gd^3+^, and Fe^3+^, which enhance contrast in CT, T1, and T2 MR modes, respectively, were substituted into the CDHA apatitic crystal structure. Preliminary results showed that the contrast enhancement in CT/T1/T2 MR modes was significant for a low amount of ionic substitution (0.25 at.% Ag^+^, 0.5 at.% Gd^3+^, and 0.25 at.% Fe^3+^). These multifunctional CDHA nanoparticles were inherently antibacterial due to Ag^+^ substitution and also showed predominantly burst release of tetracycline antibiotic. The ion-substituted CDHA nanocarriers were also biocompatible due to the total ionic substitution of 1 at.%. The antibiotic-loaded triple-ion-substituted CDHA nanoparticles with antibacterial activity and multimodal image contrast are highly suitable for clinical orthopedic applications.

## Data Availability

All datasets generated for this study are included in the manuscript and/or the supplementary files.

## Author Contributions

TS and MK conceived the idea. MK performed the material synthesis, characterization, drug release, and imaging experiments. RY planned and carried out the antibacterial and biocompatibility studies under the guidance of MD.

### Conflict of Interest Statement

The authors declare that the research was conducted in the absence of any commercial or financial relationships that could be construed as a potential conflict of interest.
